# Effect of antibiotic therapy versus no antibiotics on nonoperative management outcomes in uncomplicated appendicitis: A systematic review and meta-analysis

**DOI:** 10.1007/s00384-026-05147-1

**Published:** 2026-05-09

**Authors:** Wei-Tang Lin, Yu-Ning Huang, Jen-Hung Wang, Yun-Kuan Lin

**Affiliations:** 1 Department of Emergency Medicine, Hualien Tzu Chi Hospital, Buddhist Tzu Chi Medical Foundation, Hualien, Taiwan, No. 707, Sec. 3, Zhongyang Rd., Hualien City, Hualien County 970473 Taiwan; 2 Department of Anesthesiology, Hualien Tzu Chi Hospital, Buddhist Tzu Chi Medical Foundation, Hualien, Taiwan, No. 707, Sec. 3, Zhongyang Rd., Hualien City, Hualien County 970473 Taiwan; 3 Department of Medical Research, Hualien Tzu Chi Hospital, Buddhist Tzu Chi Medical Foundation, Hualien, Taiwan, No. 707, Sec. 3, Zhongyang Rd., Hualien City, Hualien County 970473 Taiwan

**Keywords:** Antibiotics, Uncomplicated appendicitis, Nonoperative management, Treatment success, Appendectomy during follow-up

## Abstract

**Purpose:**

Although antibiotics are a recognized alternative to appendectomy for uncomplicated appendicitis, their specific benefit over observation alone remains unclear. This systematic review and meta-analysis aimed to isolate the antibiotic effect by directly contrasting antibiotics with observation in patients receiving nonoperative management (NOM) for uncomplicated appendicitis.

**Methods:**

We systematically searched PubMed, Embase (Ovid), the Cochrane Central Register of Controlled Trials, ClinicalTrials.gov, the International Clinical Trials Registry Platform, and the International Standard Randomized Controlled Trial Number Registry from inception to October 5, 2025, to identify randomized or quasi-randomized trials comparing antibiotics versus observation (no antibiotics) for NOM. We defined treatment success at 30 days as the primary endpoint. To assess the potential risk-of-bias (ROB), the Cochrane RoB 2 instrument was employed, while the overall certainty of our findings was determined through the Grading of Recommendations Assessment, Development, and Evaluation (GRADE) framework.

**Results:**

Three trials involving 437 participants were included: two randomized controlled trials (RCTs) and one quasi-RCT. Analysis restricted to RCTs demonstrated no significant effect of antibiotics on initial treatment success (RR: 1.03, 95% CI: 0.92–1.15). Analysis across three studies showed no significant differences in recurrence (RR: 1.38, 95% CI: 0.68–2.80) or the need for appendectomy during follow-up (RR: 0.98, 95% CI: 0.66–1.47). Evidence certainty was low for the primary outcome and very low for secondary outcomes.

**Conclusion:**

Current evidence is insufficient to determine whether antibiotics provide additional benefit over observation for uncomplicated appendicitis. Larger, adequately powered trials are needed to establish the comparative effectiveness of these approaches.

**Supplementary Information:**

The online version contains supplementary material available at 10.1007/s00384-026-05147-1.

## Background

Among acute abdominal conditions requiring emergency care, appendicitis stands as one of the most frequently encountered diagnoses necessitating operative management [1]. Historically, appendectomy has served as the gold-standard therapeutic modality [2]. Contemporary research indicates that conservative treatment with antibiotics may represent a reasonable alternative for patients presenting with uncomplicated forms of the disease [3, 4], potentially reducing surgery-related complications [5]. Despite these encouraging findings, antibiotic therapy is not without limitations, such as treatment failure and recurrence [6]. As such, a recent systematic review [7] has synthesized available evidence to identify specific patient and disease characteristics that predict the success or failure of nonoperative management (NOM). Furthermore, some studies have revealed that uncomplicated appendicitis may resolve spontaneously [8], raising uncertainty about its actual benefit.

Several studies [9–12] have compared antibiotic therapy versus surgery for uncomplicated appendicitis, contributing to a growing evidence base. The recent 10-year follow-up of the landmark APPAC trial has further provided robust evidence supporting the long-term viability of antibiotic-based NOM, demonstrating sustained safety and efficacy over an extended observation period [13]. However, a critical knowledge gap persists; direct comparative evidence against observation in uncomplicated appendicitis patients without antibiotic treatment remains limited [14, 15], and existing studies have reported inconsistent findings [16]. These contrasting findings emphasize the unresolved question of whether antibiotics have real benefits over observation in uncomplicated appendicitis.

Existing systematic reviews have not specifically addressed whether antimicrobial agents provide added benefit compared to observation alone among patients receiving NOM for uncomplicated appendicitis. Therefore, we conducted this evidence synthesis to compare outcomes between antibiotic-based and observation-only approaches within NOM strategies, aiming to support clinical decision-making and identify research gaps.

## Methods

This systematic review and meta-analysis synthesized evidence from randomized controlled trials (RCTs) and quasi-RCTs comparing antimicrobial therapy versus observation (no antibiotics) in patients with uncomplicated acute appendicitis undergoing NOM. The review was conducted following the Preferred Reporting Items for Systematic Reviews and Meta-Analyses (PRISMA) 2020 guidelines [17], and our protocol was registered prospectively on PROSPERO (registration ID: CRD420251176895). The completed PRISMA checklist is available in the supplementary materials.

### Search strategy

Pertinent publications were identified by searching PubMed, Embase (Ovid), Cochrane Central Register of Controlled Trials, ClinicalTrials.gov, the WHO International Clinical Trials Registry Platform (ICTRP), and the International Standard Randomized Controlled Trial Number (ISRCTN) Registry from inception to October 5, 2025, using the keywords “appendix,” “appendicitis,” “antibiotics,” and related synonyms. Supplementary Table S1 shows the detailed search strategy. No methodological or language restrictions were applied.

To minimize publication bias, our search strategy incorporated grey literature as follows: (1) trial registries (ClinicalTrials.gov, WHO ICTRP, and ISRCTN Registry) were searched to identify unpublished or ongoing trials; (2) conference proceedings and abstracts were captured through Embase and the Cochrane Central Register of Controlled Trials; and (3) backward citation tracking was performed on all included studies and relevant systematic reviews. No additional eligible trials beyond those published in peer-reviewed journals were identified.

### Study inclusion criteria and selection process

Inclusion required studies to meet the following specifications: (1) randomized or quasi-randomized trial design; (2) participant enrollment limited to individuals with uncomplicated acute appendicitis confirmed through clinical evaluation or computed tomography (CT) scan; (3) intervention consisting of systemic antibiotic administration via oral, intravenous, intramuscular, or combination delivery routes; and (4) comparator group managed with observation in the absence of antimicrobial therapy. YNH and YKL independently performed title and abstract screening to identify potentially relevant reports, and subsequently conducted a comprehensive full-text review for eligibility confirmation.

### Outcome definitions and measurement

Evidence from a landmark randomized trial assessing antibiotic efficacy in uncomplicated acute appendicitis (CODA trial) [9] demonstrated that most individuals who failed conservative management underwent surgical intervention within 30 days following randomization. We therefore designated initial treatment success within this 30-day window as our primary outcome measure, defined uniformly across both groups as absence of appendectomy within 30 days. Studies reporting outcomes at approximately 30 days (e.g., 1 month, 4 weeks) were included when exact 30-day data were unavailable.

The secondary outcomes included recurrence of appendicitis, appendectomy during long-term follow-up, complicated appendicitis at surgery, length of hospital stay (LOS), and adverse events. Appendicitis recurrence was defined as a new episode occurring > 30 days (or a comparable threshold specified in the original trial) after inclusion, requiring surgery or antibiotic therapy, among patients without a previous history of appendectomy, and assessed up to the longest follow-up reported in each included study. Appendectomy during long-term follow-up was defined as the proportion of patients who underwent appendectomy at any time point within the same follow-up period as reported by the original study. Adverse events were defined as any unfavorable medical conditions related to the treatment, including both antibiotic-related adverse reactions and surgery-related complications.

### Data extraction

YKL and WTL performed data extraction and verification, respectively. Retrieved information included: (1) first author, (2) publication year, (3) study country, (4) study design, (5) inclusion criteria, (6) patient numbers and characteristics (e.g., age and sex), (7) intervention and control treatments, (8) treatment success, (9) appendectomy rate, (10) operative and histopathological findings, (11) LOS, (12) adverse events, (13) sources of funding, and (14) trial registration number.

In one trial [16] that presented appendectomy data exclusively as a Kaplan–Meier curve, data on 30-day treatment success were extracted from the published figure using WebPlotDigitizer version 4.8 [18]. The Kaplan–Meier curves utilized distinct markers to denote individual patient events: circles (○) represented uncensored events (i.e., patients who underwent appendectomy), while plus signs (+) indicated censored observations (i.e., patients who completed follow-up without appendectomy). The two study groups were distinguished by line type: a solid blue line for the antibiotic group and a dashed red line for the observation group.

We employed a direct enumeration approach comprising the following steps: (1) calibration of the temporal X-axis based on clearly marked grid lines at 0 and 100 days to establish an accurate time scale; (2) determination of the 30-day threshold position using proportional distance calculation (distance from 0 to 100 days multiplied by 3/10); (3) visual identification and manual counting of individual uncensored event markers (circles) occurring at or before this 30-day threshold along each treatment group's curve; (4) calculation of cumulative appendectomy events by directly tallying uncensored markers for each group. This direct counting method provided exact event counts without requiring estimation from Y-axis coordinates, thereby enhancing precision and reproducibility. To ensure data integrity, the extracted event counts were independently verified by two reviewers (YKL and WTL), and any discrepancies were resolved by consensus.

### Quality assessment

Two independent reviewers (WTL and YKL) evaluated the risk-of-bias (RoB) using the Cochrane RoB tool, version 2 [19]. The assessment framework encompassed six key domains: randomization procedures, adherence to assigned interventions, completeness of outcome data, measurement accuracy, reporting selectivity, and aggregate bias. Disagreements between reviewers were reconciled through discussion to achieve consensus. Visual representations of bias assessments were created using the Risk-of-Bias VISualization (robvis) package [20].

### Meta-analysis

Quantitative synthesis was conducted in Review Manager 5.4 (The Cochrane Collaboration, Copenhagen, 2020) employing the Mantel–Haenszel approach. We adopted a random-effects framework to accommodate expected variability across studies. Between-study heterogeneity was quantified using the I^2^ metric. For binary endpoints, we computed risk ratios (RRs) alongside their 95% confidence intervals (CIs). Given the small number of trials analyzed (fewer than 10), we omitted funnel plot construction to avoid unreliable asymmetry detection.

### Subgroup and sensitivity analyses

All included trials enrolled only adult patients, and the number of eligible studies was limited. Thus, the planned subgroup analyses according to patient population (adults versus children) and type of antibiotic regimen were not performed. For the primary outcome, meta-analyses were performed on both the RCT-only dataset and the complete dataset (including the quasi-RCT).

### Certainty of evidence assessment

To determine the strength of our findings, we applied the Grading of Recommendations Assessment, Development, and Evaluation (GRADE) approach [21]. The assessment incorporated five core components: RoB, between-study heterogeneity, indirectness of evidence, imprecision of effect estimates, and potential publication bias. Consequently, we assigned a certainty rating to each outcome, ranging from high to very low.

## Results

**Fig. 1:**
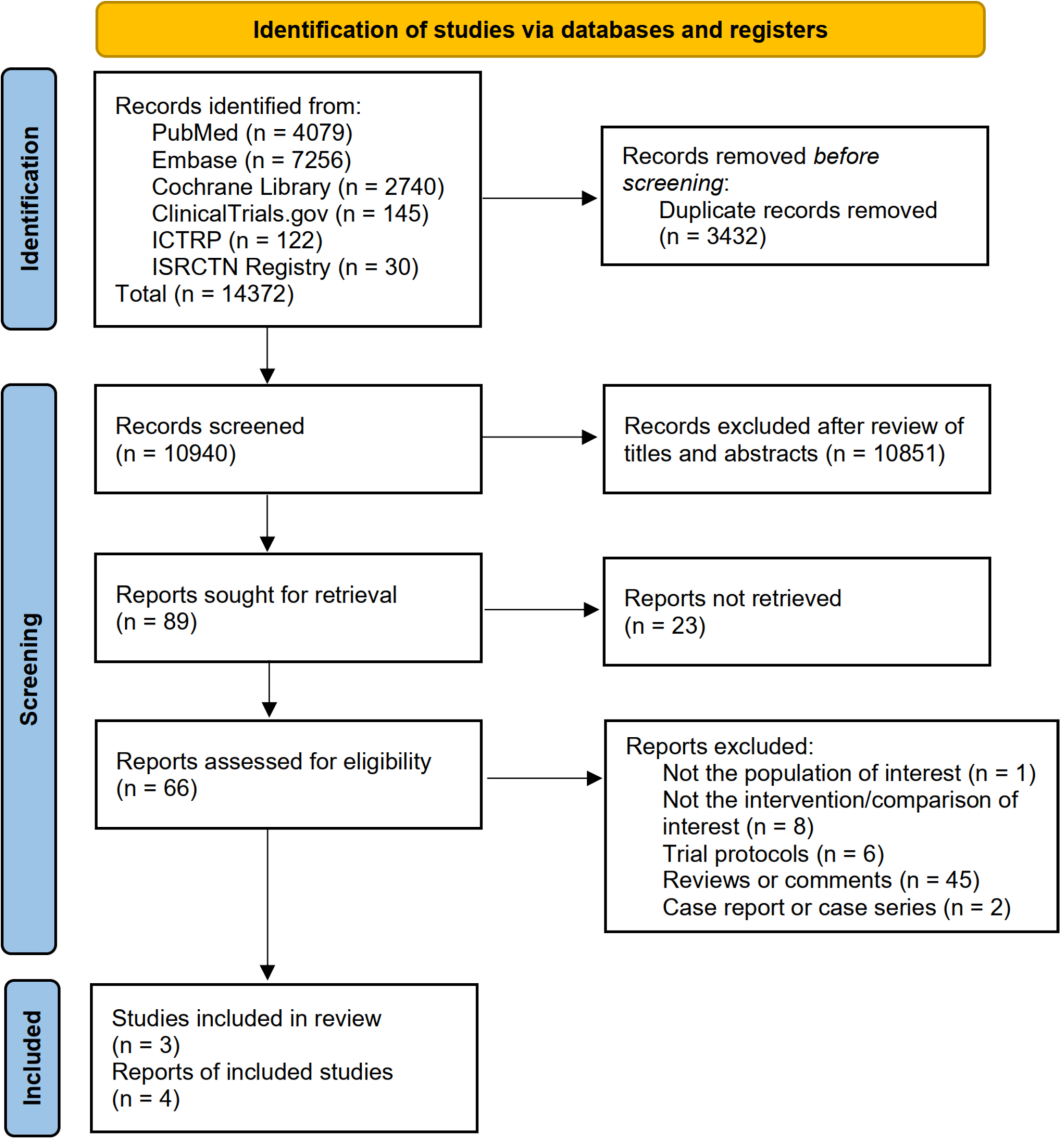
Study flowchart of preferred reporting items for systematic reviews and meta-analyses

Database searches across six platforms retrieved 14372 citations, of which 10940 unique records underwent screening after deduplication. An initial review of titles and abstracts resulted in the exclusion of 10851 records. An additional 23 records were excluded because the full-text articles were unavailable. A detailed eligibility assessment resulted in the further exclusion of 62 studies, with specific reasons documented in Fig. 1 and Supplementary Table S2. Three trials (described in four publications) met all inclusion criteria and were incorporated into the meta-analysis.

### Characteristics of the included studies

The included trials were conducted in South Korea [14], Finland [15], and Sweden [16], and all patients were adults (≥ 18 years). Two studies [14, 15] included patients with CT scan-confirmed uncomplicated acute appendicitis. Meanwhile, the third trial [16] enrolled patients with clinically diagnosed appendicitis. In the third trial, participants were identified using a previously validated model [22], which selects individuals with a high probability of appendicitis without wall necrosis or perforation, corresponding to clinically defined uncomplicated appendicitis. Based on the model’s original validation, these individuals were expected to have high rates of successful conservative treatment with antibiotics. The choice and length of antibiotic protocols varied between trials. Study characteristics are detailed in Table 1.

**Table 1:** Characteristics of the included studies

Study	Country	Study design	Inclusion criteria	Group	Number of patients	Treatments	Outcome measures	Sources of funding	Trial registration number
Park et al., 2017	South Korea	Single-blind RCT	• Age 18–70 years • CT-confirmed uncomplicated appendicitis (diameter 6.1–11.0 mm) • Exclusions: appendicolith, perforation, free fluid, or abscess/mass	Intervention	121	• Cefmetazole 2 g + metronidazole 1.5g IV daily × 3–4 days • Supportive care + 24-h fasting period	• Primary: total treatment failure (initial treatment failure at 1 month + recurrence) • Secondary: appendectomy rate, operative findings, lab findings (WBC, CRP), adverse events, LOS, costs	Hallym University Medical Center	KCT0000124
				Control	124	• Placebo • Supportive care + 24-h fasting period			
Salminen et al., 2022	Finland	Double-blind RCT	• Age 18–60 years • CT-confirmed uncomplicated appendicitis (diameter > 6 mm) • Exclusions: appendicolith, perforation, abscess, or tumor	Intervention	35	• Ertapenem 1 g IV daily × 3 days → levofloxacin 500 mg daily + metronidazole 500 mg TID × 4 days (oral) • Supportive care	• Primary: resolution without surgery within 10 days • Secondary: appendectomy rate (10 days, 30 days, 1 year, 3 years), 3-year recurrence, operative findings, lab findings, pain, adverse events, LOS, sick leave, symptoms, quality of life	Sigrid Jusélius Foundation, Academy of Finland, Mary and Georg C. Ehrnrooth Foundation, The Finnish Medical Foundation, and Orion Research Foundation	EudraCT 2015—003634—26, NCT03234296
				Control	31	• Placebo • Supportive care			
Iresjö et al., 2024	Sweden	Quasi-RCT	• Age 18–60 years • Clinically diagnosed appendicitis • Laboratory criteria: WBC < 13,000/µL and CRP < 60 mg/L	Intervention	69	• Piperacillin/tazobactam 4g/500mg IV q8h → ciprofloxacin 500 mg BID + metronidazole 400 mg TID × 8–10 days (oral) • Supportive care	• Primary: appendectomy rate (up to 1200 days) • Secondary: operative findings, LOS	The Swedish government and the county councils, under the ALF agreement	EudraCT 2018—01578—71, NCT03985514
				Control	57	• Observation without antibiotics • Supportive care			

BID: twice daily; CRP: C-reactive protein; CT: computed tomography; IV: intravenous; LOS: length of hospital stay; q8h: every 8 h; TID: three times daily; WBC: white blood cell count

### Quality assessment

RoB was assessed using the Cochrane RoB 2.0 tool. While assessment was performed for each outcome, RoB judgments were consistent across outcomes, given similar measurement approaches and data availability.

### Domain-level RoB summary

Randomization process: Both RCTs demonstrated a low RoB with appropriate sequence generation and allocation concealment. The quasi-RCT had a high risk as allocation by alternating months made the sequence predictable.

Deviations from interventions: Park et al. had a low risk with all patients following assigned interventions and a complete intention-to-treat analysis. Salminen et al. had some concerns due to five post-randomization withdrawals violating intention-to-treat principles. Iresjö et al. had a high risk due to substantial post-randomization withdrawal.

Missing outcome data: Park et al. and Salminen et al. had a low risk with minimal attrition. Iresjö et al. had a high risk due to substantial missingness post-randomization with unequal final group sizes (69 vs 57), suggesting differential missing data between groups.

Outcome measurement: Park et al. and Salminen et al. had some concerns due to a lack of outcome assessor blinding (single-blind design in Park et al.; differential side effects potentially unblinding in Salminen et al.). Iresjö et al. had a high risk due to a lack of blinding combined with potential subjective influence on clinical decisions.

Selective reporting: Salminen et al. had a low risk with outcomes as prespecified. Park et al. had some concerns as the protocol specified pain as the primary outcome, but this was not reported in the publication. Iresjö et al. had some concerns due to the absence of a detailed protocol for outcome measurement and analysis.

**Fig. 2:**
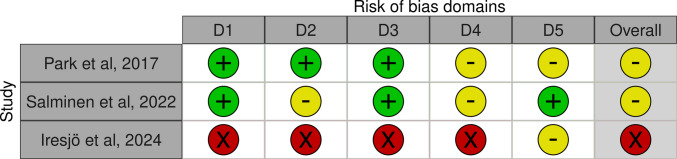
Risk-of-bias traffic-light plot. D1: Bias from randomization; D2: Bias from protocol deviations; D3: Bias from incomplete outcome data; D4: Bias in outcome assessment; D5: Bias in selective reporting. Green (+) = low risk-of-bias; yellow (-) = moderate risk-of-bias; red (X) = high risk-of-bias

**Fig. 3:**
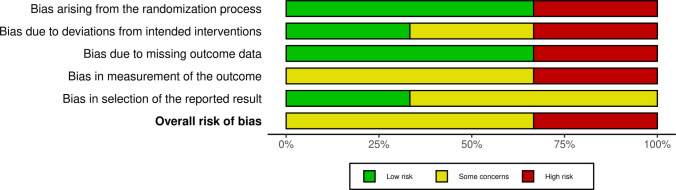
Risk-of-bias summary plot

Overall RoB: Both RCTs had some concerns overall (primarily blinding limitations and, for Park et al., selective reporting; for Salminen et al., post-randomization withdrawals). The quasi-RCT had a high overall risk (predictable allocation, post-randomization withdrawals, substantial attrition, lack of blinding). RoB assessments are presented in Fig. 2 (traffic-light plot) and Fig. 3 (summary plot).

### Primary outcome: initial treatment success

Two RCTs reported treatment success rates at 1 month [14] and 30 days [15], respectively. Meanwhile, a quasi-RCT [16] provided a Kaplan–Meier curve depicting the time course of appendectomy. In this quasi-RCT, data on the 30-day treatment success were extracted from the figure with the help of WebPlotDigitizer. Initial analysis, including all three studies, showed substantial heterogeneity (I^2^ = 87%), primarily attributable to the quasi-RCT. Consequently, we prioritized the RCT-only analysis to provide the most robust estimate, followed by the all-studies analysis.

### Meta-analysis restricted to RCTs

**Fig. 4:**
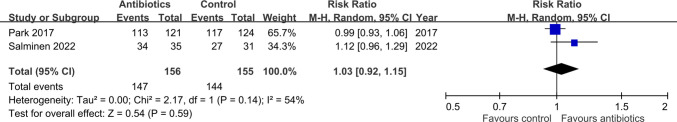
Meta-analysis of randomized controlled trials for initial treatment success

Analysis of the two RCTs demonstrated no significant difference between antibiotics and observation on initial treatment success (RR: 1.03, 95% CI: 0.92–1.15, I^2^ = 54%; Fig. 4).

### Meta-analysis including all studies

**Fig. 5:**
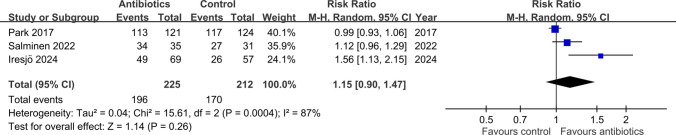
Meta-analysis of all studies for initial treatment success

When the quasi-randomized trial was included, the pooled estimate remained non-significant (RR: 1.15, 95% CI: 0.90–1.47), though substantial heterogeneity was observed (I^2^ = 87%; Fig. 5).

### Secondary outcomes: recurrence of appendicitis and appendectomy during long-term follow-up

**Fig. 6:**
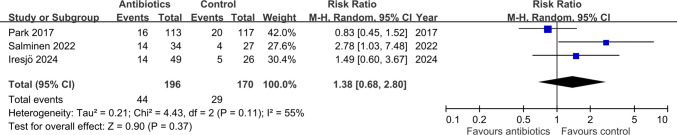
Meta-analysis of all studies for appendicitis recurrence

**Fig. 7:**
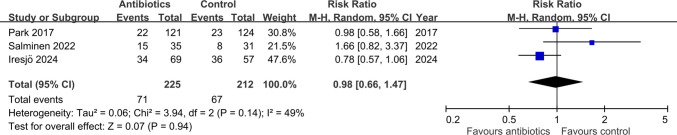
Meta-analysis of all studies for the need for appendectomy during long-term follow-up

Regarding the duration of follow-up for long-term outcomes, one RCT [15], in its subsequent follow-up publication [23], reported data at the prespecified 3-year time point. Another RCT [14] showed that the median follow-up was 19 months. The quasi-RCT [16] presented details about treatment outcomes up to the last follow-up visit, with a maximum duration of 1200 days. Pooled analysis showed no significant differences in appendicitis recurrence between groups (RR: 1.38, 95% CI: 0.68–2.80, I^2^ = 55%; Fig. 6) or need for appendectomy during long-term follow-up (RR: 0.98, 95% CI: 0.66–1.47, I^2^ = 49%; Fig. 7). However, CIs were wide, reflecting imprecision due to few events.

### Secondary outcomes: intraoperative findings of complicated appendicitis, hospital stay duration, and adverse events

Quantitative meta-analysis was not feasible for complicated appendicitis at surgery, hospital stay duration, and adverse events due to heterogeneous reporting timeframes and definitions. A systematic narrative synthesis of these findings is presented below, with detailed data summarized in Table 2.

**Table 2:** Secondary outcomes: intraoperative findings of complicated appendicitis, hospital stay duration, and adverse events

Study	Group	Intraoperative findings of complicated appendicitis, n/N (%)	Hospital stay duration	Adverse events
Park et al., 2017	Intervention	6/121 (5.0)	3.7 (1.3) days, mean (SD)	Surgical complications (n = 5): wound infection (n = 4), intra-abdominal fluid (n = 1); group allocation not specified
	Control	7/124 (5.6)	3.1 (1.3) days, mean (SD)	
Salminen et al., 2022	Intervention	1/35 (2.9)	55.5 (50–59) hours, median (IQR)^a^	Antibiotic group: 8 adverse drug reactions; Control group: 1 incisional hematoma (self-resolved)
	Control	2/31 (6.5)	52.6 (48.5–61.8) hours, median (IQR)^a^	
Iresjö et al., 2024	Intervention	10/69 (14.5)	2.1 (1.2) days, mean (SD)	Antibiotic group: 1 patient with mild antibiotic side effects, no treatment interruption
	Control	7/57 (12.3)	2.0 (1.2) days, mean (SD)	

^a^Data on LOS within 10 days

Individual trial comparisons revealed no statistically significant differences in the incidence of intraoperative complicated appendicitis between groups: Park et al. reported 5.0% (6/121) vs. 5.6% (7/124) (RR: 0.88; 95% CI: 0.30–2.54); Salminen et al. reported 2.9% (1/35) vs. 6.5% (2/31) (RR: 0.44; 95% CI: 0.04–4.65); and Iresjö et al. reported 14.5% (10/69) vs. 12.3% (7/57) (RR: 1.18; 95% CI: 0.48–2.90). Notably, all CIs crossed the null with few events and inconsistent effect directions. Moreover, variation in follow-up periods (initial hospitalization to 19 months) limited comparability.

Trials reported similar stay durations despite varied measurement approaches and reporting timeframes. Salminen et al. reported medians of 55.5 versus 52.6 h specifically within a 10-day window. In contrast, the remaining two trials did not clearly define the measurement period: Park et al. observed a 0.6-day difference (3.7 vs. 3.1 days), and Iresjö et al. noted a minimal 0.1-day difference (2.1 vs. 2.0 days) over an undefined duration. Despite these heterogeneous definitions, the numerical differences between groups remained minimal across all studies, suggesting no substantial difference in hospital stay duration between antibiotic-based and observation-based management, though heterogeneous measurement approaches limit definitive conclusions.

All trials reported low adverse event rates. Park et al. reported 5 surgical complications without group specification. Salminen et al. reported 8 antibiotic-related reactions versus 1 control-group complication. Iresjö et al. reported 1 mild antibiotic side effect. No serious safety signals were identified for either approach.

### Certainty of evidence assessment

The GRADE approach was applied to assess the quality of evidence for outcomes that could be pooled in the meta-analysis.

For the primary outcome, evidence certainty was assessed based on the RCT-only analysis and rated as low, with downgrading due to RoB and inconsistency (Supplementary Table S3). For secondary outcomes (appendicitis recurrence and need for appendectomy during long-term follow-up), evidence certainty was rated as very low based on all three studies, with downgrading primarily due to RoB and imprecision (Supplementary Table S3).

## Discussion

Our findings suggest that antibiotics may provide limited benefit over observation alone regarding short-term treatment success in conservative management of uncomplicated appendicitis. Similarly, for secondary outcomes, no significant differences were observed in either meta-analyzed outcomes (appendicitis recurrence and need for appendectomy during follow-up) or outcomes assessed through narrative synthesis (operative findings of complicated disease and hospitalization duration). Importantly, the GRADE assessment rated the certainty of the evidence as low for the primary outcome and very low for the secondary outcomes. The small number of trials (n = 3) with limited sample sizes (total n = 437) precludes definitive conclusions. Therefore, current evidence remains insufficient to determine whether antibiotics provide additional benefit over observation in the conservative management of uncomplicated appendicitis.

Analysis, including the quasi-randomized trial conducted by Iresjö et al. [16], revealed substantial statistical heterogeneity (I^2^ = 87%). This trial differed from the other two in patient selection and study design. In particular, it enrolled patients with clinically diagnosed appendicitis, with only approximately 40% undergoing a CT scan. In contrast, the remaining two trials [14, 15] required CT confirmation of uncomplicated appendicitis and excluded patients with appendicolith. Therefore, incomplete exclusion of appendicolith-positive cases in Iresjö's study could have contributed to the higher initial treatment failure rate observed in that trial. This interpretation is supported by the CODA trial [9], which demonstrated that the presence of an appendicolith was associated with an increased risk of nonoperative treatment failure [24]. In addition, the design limitations in the Iresjö trial, including the absence of allocation concealment and placebo, may have increased the risk among patients in the control group undergoing appendectomy, leading to outcomes that differed from those in the other two trials.

Beyond statistical heterogeneity, the diagnostic difference (CT-confirmed versus clinically diagnosed appendicitis) may act as an important effect modifier influencing treatment effectiveness. Specifically, meta-analysis of the two RCTs requiring CT-confirmed diagnosis demonstrated minimal difference between antibiotics and observation (RR: 1.03, 95% CI: 0.92–1.15, I^2^ = 54%), whereas the quasi-randomized trial using clinical diagnosis showed an apparent benefit favoring antibiotics (RR: 1.56, 95% CI: 1.13–2.15). This contrast suggests that diagnostic stringency may alter treatment effects through patient selection: CT confirmation enables rigorous identification of truly uncomplicated appendicitis, selecting patients who respond similarly to conservative management regardless of antibiotic use, while clinical diagnosis alone may inadvertently include patients with subclinical complicated features who benefit more from antibiotic therapy. However, this difference is confounded with study design quality (the clinically diagnosed cohort was also the only quasi-randomized trial), precluding definitive attribution to diagnostic criteria. Nevertheless, the substantial difference in effect estimates suggests diagnostic approach may influence treatment effects. Therefore, future trials should stratify outcomes by imaging confirmation status to clarify whether diagnostic criteria independently modify treatment effectiveness.

Despite these sources of heterogeneity, we included the quasi-randomized trial to ensure a comprehensive synthesis of all available comparative evidence. Indeed, with only three trials directly addressing this specific comparison, excluding published trials without a priori justification may risk presenting an incomplete evidence base. Furthermore, systematic review methodological guidelines (e.g., Cochrane Handbook) [19] acknowledge that quasi-randomized evidence may be included in evidence synthesis when the literature is limited, with appropriate consideration of methodological limitations. Therefore, we addressed the resulting heterogeneity by designating RCT-only analysis as the primary analysis, while using the all-studies analysis to demonstrate that conclusions remain consistent (no significant benefit of antibiotics) regardless of whether the quasi-RCT is included or excluded. Notably, the substantial reduction in heterogeneity when restricting to RCTs (I^2^ from 87 to 54%) validates this analytical strategy.

Based on evidence from studies of uncomplicated diverticulitis [25, 26], some acute intra-abdominal inflammatory conditions may resolve without antibiotics. In the systematic reviews and meta-analyses performed by Dichman et al. [27] and Bonito et al. [28], observation alone did not increase complications, recurrence, or the need for intervention. Considering the shared pathophysiology of diverticulitis [29] and appendicitis [30] (i.e., localized inflammation secondary to luminal obstruction), a subset of uncomplicated appendicitis cases may similarly follow a self-limiting course. Therefore, cautious patient selection using clinical, laboratory [31], or imaging criteria [32] could guide conservative management, reduce unnecessary antibiotic exposure, and inform future trial design.

In addition, decreasing antibiotic use in such patients may confer broader benefits, including reduced risk of drug-related adverse events and preservation of gut microbiota [33], and it may mitigate the emergence and spread of antimicrobial resistance [34]. Therefore, these considerations support judicious antibiotic use in uncomplicated appendicitis, where clinically appropriate.

To the best of our knowledge, this is the first systematic review and meta-analysis specifically designed to isolate the effect of antibiotics by comparing them directly against observation for uncomplicated appendicitis. Unlike existing high-level evidence [3, 4] that primarily contrasts antibiotics with surgery, our study addresses the distinct clinical question of whether antibiotics are mandatory within a nonoperative framework. While the current paucity of trials limits the overall certainty of the evidence, this study provides the most comprehensive synthesis of available data to date. Consequently, these findings identify a critical knowledge gap, providing a necessary foundation for clinical decision-making and the design of future robust trials.

Several limitations warrant acknowledgment. First, the small number of included trials and total participants may have limited statistical power, increasing the risk of type II error. Second, certain methodological shortcomings in the included trials may have influenced the robustness and certainty of the synthesized evidence. Third, the exclusive focus on adult participants across all studies limits generalizability to children and adolescents.

Future studies should include larger, multicenter trials to increase statistical power and enhance external validity. Moreover, future research may consider excluding patients with appendicolith to reduce heterogeneity and better evaluate the efficacy of conservative treatment strategies. Additionally, appropriately designed and ethically approved studies, including pediatric populations, could also be carried out to assess the safety and efficacy of antibiotic management in younger patients. Encouragingly, ongoing RCTs [35, 36] are currently investigating antibiotic therapy versus observation for uncomplicated appendicitis, and their results can provide additional evidence to further validate optimal management strategies.

## Conclusions

While NOM is an established strategy for uncomplicated appendicitis, the incremental benefit of antibiotics over observation remains a subject of debate. The current study evaluated the effects of antibiotic therapy versus observation on NOM outcomes. Our results indicate that current evidence is insufficient to definitively establish whether antibiotic treatment provides a significant clinical advantage in improving initial treatment success or reducing recurrence and the need for appendectomy during follow-up. This uncertainty reflects the limited evidence base, with only three small trials (n = 437) available for analysis. Future research priorities should include large, methodologically rigorous RCTs capable of definitively resolving whether antibiotics provide a tangible clinical benefit in the NOM of uncomplicated appendicitis.

## Supplementary Information

Below is the link to the electronic supplementary material.

Supplementary file1 (DOCX 45 KB)

## Data Availability

The datasets used and/or analyzed during the current study are available from the corresponding author upon reasonable request.
