# Supplementary material for: Effect of antibiotic therapy versus no antibiotics on nonoperative management outcomes in uncomplicated appendicitis: A systematic review and meta-analysis
Source: Int J Colorectal Dis. 2026 May 9;41(1):110. doi: 10.1007/s00384-026-05147-1 (PMC13323108; doi:10.1007/s00384-026-05147-1)
Supplement: Supplementary file 1 — Supplementary file1 (DOCX 45 KB) [file 384_2026_5147_MOESM1_ESM.docx]

**Supplementary information**

Title: Effect of antibiotic therapy versus no antibiotics on nonoperative management outcomes in uncomplicated appendicitis: A systematic review and meta-analysis

Journal: International Journal of Colorectal Disease

Authors: Wei-Tang Lin, Yu-Ning Huang, Jen-Hung Wang, Yun-Kuan Lin*

*Corresponding author: Department of Emergency Medicine, Hualien Tzu Chi Hospital, Buddhist Tzu Chi Medical Foundation, Hualien, Taiwan; yklin1129@gmail.com

| Supplementary Contents | Contents | Page |
| --- | --- | --- |
| Table S1 | Search Strategy Modified in PubMed, Embase, Cochrane Central Register of Controlled Trials, ClinicalTrials.gov, ICTRP, and ISRCTN Registry | 2-3 |
| Table S2 | List of Excluded Studies during Full-Text Screening | 4-10 |
| Table S3 | Grading of Recommendations Assessment, Development, and Evaluation (GRADE) Analysis | 11-12 |

**Table S1.** Search Strategy Modified in PubMed (a), Embase (b), Cochrane Central Register of Controlled Trials (c), ClinicalTrials.gov (d), ICTRP (e), and ISRCTN Registry (f)

1. **Search strategy in PubMed (to present; search date: 2025/10/05)**

| **#** | **Search strategy** |
| --- | --- |
| 1 | (appendicitis) OR (appendix) |
| 2 | (antibiotic*) OR (antibiotic) OR (antimicrobial*) OR (antimicrobial) OR (anti-microbial*) OR (anti-microbial) OR (antibacterial*) OR (antibacterial) OR (anti-bacterial*) OR (anti-bacterial) OR (anti infective*) OR (anti infective) OR (antiinfective*) OR (antiinfective) OR (anti-infective*) OR (anti-infective) |
| 3 | 1 AND 2 |

1. **Search strategy in Embase (via Ovid, to present; search date: 2025/10/05)**

| **#** | **Search strategy** |
| --- | --- |
| 1 | (appendicitis or appendix).mp. |
| 2 | appendicitis/ or appendix/ |
| 3 | 1 or 2 |
| 4 | (antibiotic* or antibiotic or antimicrobial* or antimicrobial or anti-microbial* or anti-microbial or antibacterial* or antibacterial or anti-bacterial* or anti-bacterial or "anti infective*" or "anti infective" or antiinfective* or antiinfective or anti-infective* or anti-infective).mp. |
| 5 | antibiotic agent/ or antiinfective agent/ |
| 6 | 4 or 5 |
| 7 | 3 and 6 |

1. **Search strategy in Cochrane Central Register of Controlled Trials Database (CENTRAL; to present; search date: 2025/10/05)**

| **#** | **Search strategy** |
| --- | --- |
| 1 | appendicitis OR appendix |
| 2 | MeSH descriptor: [Appendicitis] explode all trees |
| 3 | MeSH descriptor: [Appendix] explode all trees |
| 4 | #1 OR #2 OR #3 |
| 5 | antibiotic* OR antibiotic OR antimicrobial* OR antimicrobial OR anti-microbial* OR anti-microbial OR antibacterial* OR antibacterial OR anti-bacterial* OR anti-bacterial OR antiinfective* OR antiinfective OR anti-infective* OR anti-infective |
| 6 | anti infective* |
| 7 | anti infective |
| 8 | MeSH descriptor: [Anti-Bacterial Agents] explode all trees |
| 9 | MeSH descriptor: [Anti-Infective Agents] explode all trees |
| 10 | #5 OR #6 OR #7 OR #8 OR #9 |
| 11 | #4 AND #10 |

1. **Search strategy in ClinicalTrials.gov (to present; search date: 2025/10/05)**

| **#** | **Search strategy** |
| --- | --- |
| 1 | ((appendicitis) OR (appendix)) AND ((antibiotic*) OR (antibiotic) OR (antimicrobial*) OR (antimicrobial) OR (anti-microbial*) OR (anti-microbial) OR (antibacterial*) OR (antibacterial) OR (anti-bacterial*) OR (anti-bacterial) OR (anti infective*) OR (anti infective) OR (antiinfective*) OR (antiinfective) OR (anti-infective*) OR (anti-infective)) |

1. **Search strategy in ICTRP (to present; search date: 2025/10/05)**

| **#** | **Search strategy** |
| --- | --- |
| 1 | ((appendicitis) OR (appendix)) AND ((antibiotic*) OR (antibiotic) OR (antimicrobial*) OR (antimicrobial) OR (anti-microbial*) OR (anti-microbial) OR (antibacterial*) OR (antibacterial) OR (anti-bacterial*) OR (anti-bacterial) OR (anti infective*) OR (anti infective) OR (antiinfective*) OR (antiinfective) OR (anti-infective*) OR (anti-infective)) |

1. **Search strategy in ISRCTN Registry (to present; search date: 2025/10/05)**

| **#** | **Search strategy** |
| --- | --- |
| 1 | ((appendicitis) OR (appendix)) AND ((antibiotic*) OR (antibiotic) OR (antimicrobial*) OR (antimicrobial) OR (anti-microbial*) OR (anti-microbial) OR (antibacterial*) OR (antibacterial) OR (anti-bacterial*) OR (anti-bacterial) OR (anti infective*) OR (anti infective) OR (antiinfective*) OR (antiinfective) OR (anti-infective*) OR (anti-infective)) |

**Table S2.** List of Excluded Studies during Full-Text Screening

| **Study** | **Reason for Exclusion** |
| --- | --- |
| DALES HC. Streptomycin and the tetracyclines in acute appendicitis. *Ulster Med J*. 1954;23(1):61-62. | Not the population of interest |
| Pátková B, Svenningsson A, Almström M, et al. Long-Term Outcome of Nonoperative Treatment of Appendicitis. *JAMA Surg*. 2023;158(10):1105-1106. doi:10.1001/jamasurg.2023.2756 | Not the intervention/comparison of interest |
| Puputti J, Suominen JS, Luoto T, et al. A randomized, controlled multicenter feasibility pilot trial on imaging confirmed uncomplicated acute appendicitis: Appendectomy vs. symptomatic treatment in pediatric patients (the APPSYPP) trial study protocol. *Contemp Clin Trials*. 2022;123:106970. doi:10.1016/j.cct.2022.106970 | Not the intervention/comparison of interest |
| Wu J, Jiang H, Li S, et al. Optimising the treatment for uncomplicated acute appendicitis (OPTIMA trial): a protocol for a multicentre, randomised, double-blinded placebo-controlled study. *BMJ Open*. 2022;12(5):e057793. Published 2022 May 2. doi:10.1136/bmjopen-2021-057793 | Not the intervention/comparison of interest |
| Knaapen M, Van der Lee JH, Gaillard EL, et al. Non-operative treatment of children with simple appendicitis: long-term follow-up (5 years) in a prospective cohort study. *Br J Surg*. 2021;108(3):e99-e100. doi:10.1093/bjs/znaa052 | Not the intervention/comparison of interest |
| Auer S. Les antibiotiques pour une appendicite non compliquée : une alternative envisageable ?. *Rev Med Suisse*. 2019;15(634):180. | Not the intervention/comparison of interest |
| Non-operative Management of Early Appendicitis in Children [Internet]. 2012. Available from: https://clinicaltrials.gov/study/NCT01718275. | Not the intervention/comparison of interest |
| Farahnak M, Talaei-Khoei M, Gorouhi F, Jalali A, Gorouhi F. The Alvarado score and antibiotics therapy as a corporate protocol versus conventional clinical management: randomized controlled pilot study of approach to acute appendicitis. *Am J Emerg Med*. 2007;25(7):850-852. doi:10.1016/j.ajem.2007.01.012 | Not the intervention/comparison of interest |
| Simpson ET, Corbett H. Antibiotic use in appendicitis. ANZ journal of surgery. 2002;72 Suppl:A72. | Not the intervention/comparison of interest |
| Sippola S, Grönroos J, Sallinen V, et al. A randomised placebo-controlled double-blind multicentre trial comparing antibiotic therapy with placebo in the treatment of uncomplicated acute appendicitis: APPAC III trial study protocol. *BMJ Open*. 2018;8(11):e023623. Published 2018 Nov 3. doi:10.1136/bmjopen-2018-023623 | Trial protocol |
| Optimized Treatment for Uncomplicated Acute Appendicitis; Active Observation With or Without Antibiotic Treatment [Internet]. 2018. Available from: https://clinicaltrials.gov/study/NCT03985514. | Trial protocol |
| Kct. Conservative management without antibotics in patient wtih equivocal apppendicitis. https://trialsearch.who.int/Trial2.aspx?TrialID=KCT0000124. 2011. | Trial protocol |
| Lund H, Haijanen J, Suominen S, et al. A randomized double-blind noninferiority clinical multicenter trial on oral moxifloxacin versus placebo in the outpatient treatment of uncomplicated acute appendicitis: APPAC IV study protocol. *Scand J Surg*. 2025;114(1):3-12. doi:10.1177/14574969241293018 | Trial protocol |
| Vanhatalo S, Munukka E, Sippola S, et al. Prospective multicentre cohort trial on acute appendicitis and microbiota, aetiology and effects of antimicrobial treatment: study protocol for the MAPPAC (Microbiology APPendicitis ACuta) trial. *BMJ Open*. 2019;9(9):e031137. Published 2019 Sep 6. doi:10.1136/bmjopen-2019-031137 | Trial protocol |
| ChiCtr. Randomized controlled trial for laxative therapy in the treatment of uncomplicated appendicitis. https://trialsearch.who.int/Trial2.aspx?TrialID=ChiCTR1900027324. 2019. | Trial protocol |
| de Costa A, Reismann M, Carvalho N. The Human Appendix and Antibiotics: Choice and Beneficence. *ANZ J Surg*. Published online September 30, 2025. doi:10.1111/ans.70326 | Review |
| Mott T, Gray C. Antibiotic Treatment for Uncomplicated Acute Appendicitis in Children. *Am Fam Physician*. 2025;111(1):75-76. | Review |
| Moris D, Pappas T. Elective Appendectomy Following Antibiotics for Appendicitis. *JAMA Surg*. 2024;159(6):599-600. doi:10.1001/jamasurg.2023.8036 | Comment |
| George PE, Howard DH. Nonoperative management in acute, uncomplicated appendicitis. *Surgery*. 2024;175(5):1460-1461. doi:10.1016/j.surg.2024.01.017 | Comment/Editorial |
| Fall F, Berman L. Antibiotic Use in Gangrenous, Suppurative, or Exudative Appendicitis. *JAMA Surg*. 2024;159(5):517-518. doi:10.1001/jamasurg.2023.7776 | Comment |
| Moran B. Conservative management of appendicitis: general adoption may be jumping the gun. *Br J Surg*. 2023;110(12):1601-1602. doi:10.1093/bjs/znad334 | Comment |
| Hu B, Ali FS, Huanfei D. Non-invasive interventional management of acute uncomplicated appendicitis. *Asian J Surg*. 2023;46(11):5018. doi:10.1016/j.asjsur.2023.06.045 | Comment/Letter |
| Germer CT, Reibetanz J. Antibiotische Therapie vs. Placebo bei Patienten mit CT-bestätigter unkomplizierter Appendizitis: Ergebnisse der randomisierten APPAC-III-Studie [Antibiotic treatment vs. placebo in patients with uncomplicated appendicitis: results of the randomized APPAC III study]. *Chirurgie (Heidelb)*. 2023;94(3):274-275. doi:10.1007/s00104-023-01835-8 | Comment |
| Minneci PC, Talan DA, Deans KJ. Result Interpretation in Nonoperative Management of Uncomplicated Appendicitis. *Pediatrics*. 2022;150(5):e2022059372. doi:10.1542/peds.2022-059372A | Comment/Letter |
| Salminen P. RCT Evidence Supports Incorporating Nonoperative Management Option for Uncomplicated Acute Appendicitis-How to Implement This in Clinical Practice?. *JAMA Surg*. 2022;157(7):607-608. doi:10.1001/jamasurg.2022.1555 | Comment |
| Assimakopoulos SF, Maroulis I, Triantos C, Marangos M. Oral Antibiotics for Uncomplicated Acute Appendicitis: The Role of Extended-Spectrum Beta-Lactamase Risk Factor Stratification. *Gastroenterology Res*. 2021;14(6):311-312. doi:10.14740/gr1489 | Comment/Editorial |
| Farooq A, Rouleau-Fournier F, Brown C. Antibiotics alone in the treatment of appendicitis. *CMAJ*. 2021;193(21):E769. doi:10.1503/cmaj.202777 | Review |
| Minneci PC, Deans KJ. Evolving Issues in the Use of Antibiotics for the Treatment of Uncomplicated Appendicitis. *JAMA*. 2021;325(4):351-352. doi:10.1001/jama.2020.23607 | Comment/Editorial |
| Becker P, Schilling D. Antibiotic Therapy. *Dtsch Arztebl Int*. 2021;118(1-2):10. doi:10.3238/arztebl.m2021.0029 | Comment/Letter |
| Napolitano LM. Efficacy of antibiotics in acute appendicitis treatment. *Am J Surg*. 2020;219(4):690. doi:10.1016/j.amjsurg.2019.06.016 | Comment/Letter |
| Jacobs D. Antibiotics for Appendicitis - Proceed with Caution. *N Engl J Med*. 2020;383(20):1985-1986. doi:10.1056/NEJMe2029126 | Comment/Editorial |
| Salminen P, Grönroos J. Questioning the Higher Abscess Rate and Overall Cost of Care Associated With Nonoperative Management of Uncomplicated Acute Appendicitis. *JAMA Surg*. 2019;154(8):784. doi:10.1001/jamasurg.2019.1161 | Comment/Letter |
| Podda M, Di Saverio S, Cillara N, Gerardi C. Randomized clinical trial of antibiotic therapy for uncomplicated appendicitis: Time to change the goal of our research?. *Int J Surg*. 2017;48:264-265. doi:10.1016/j.ijsu.2017.11.035 | Comment/Letter |
| Bonadio WA. Nonoperative Treatment of Appendicitis. *JAMA Pediatr*. 2017;171(11):1125. doi:10.1001/jamapediatrics.2017.2937 | Comment/Letter |
| Minneci PC, Deans KJ. Nonoperative Treatment of Appendicitis. *JAMA Pediatr*. 2017;171(11):1126-1127. doi:10.1001/jamapediatrics.2017.2940 | Comment/Letter |
| Zolot J. Antibiotics are a Treatment Alternative for Acute Appendicitis in Children. *Am J Nurs*. 2017;117(8):57. doi:10.1097/01.NAJ.0000521977.69212.30 | Comment |
| Kharbanda AB, Schmeling DJ. Are Antibiotics a Feasible Therapeutic Option for Appendicitis?. *Ann Emerg Med*. 2017;70(1):15-17. doi:10.1016/j.annemergmed.2016.12.001 | Comment/Editorial |
| Werner S, Grock A, Mason J. Antibiotics Only for Appendicitis?. *Ann Emerg Med*. 2017;70(1):12-14. doi:10.1016/j.annemergmed.2017.05.003 | Comment |
| Minneci PC, Deans KJ. Nonoperative Treatment of Appendicitis. *JAMA Pediatr*. 2017;171(11):1126-1127. doi:10.1001/jamapediatrics.2017.2940 | Comment/Letter |
| Kirby A, Burke D, Hobson R. Outcomes After Surgical and Antibiotic Treatment of Appendicitis. *World J Surg*. 2017;41(1):349-350. doi:10.1007/s00268-016-3678-8 | Comment/Letter |
| Talan DA, Moran GJ, Saltzman DJ. Nonoperative Management of Appendicitis: Avoiding Hospitalization and Surgery. *J Am Coll Surg*. 2017;224(5):994. doi:10.1016/j.jamcollsurg.2017.01.001 | Comment/Letter |
| Svensson J. Antibiotic treatment of appendicitis. *J Pediatr*. 2016;176:221-224. doi:10.1016/j.jpeds.2016.06.074 | Comment |
| Reibetanz J, Germer CT. Antibiotische Therapie bei akuter, unkomplizierter Appendizitis [Antibiotic Treatment of acute, uncomplicated appendicitis]. *Chirurg*. 2016;87(3):254. doi:10.1007/s00104-016-0166-5 | Comment |
| Lubrano J, Menahem B. The NOTA (Non Operative Treatment for Acute Appendicitis) Study: One Step Beyond or Nearby?. *Ann Surg*. 2016;263(2):e22. doi:10.1097/SLA.0000000000000921 | Comment/Letter |
| *Clinical Pharmacist*, CP, January 2016, Vol 8, No 1;8(1):DOI:10.1211/PJ.2015.20200339 | Comment |
| Nieuwenhuijs V. Eerst antibiotica veilig bij volwassenen met acute ongecompliceerde appendicitis. Ned Tijdschr Geneeskd. Published 2016. Accessed October 28, 2025. https://www.ntvg.nl/artikelen/eerst-antibiotica-veilig-bij-volwassenen-met-acute-ongecompliceerde-appendicitis | Comment/News |
| Waxman BP. Treating uncomplicated appendicitis without surgery: will computer tomography scans and antibiotics triumph over clinical acumen and surgical dogma?. *ANZ J Surg*. 2015;85(11):800. doi:10.1111/ans.13326 | Comment |
| Watkins RR. Antibiotics for Acute Appendicitis. Infectious disease alert. 2015;34(11):121‐2. | Comment |
| Mason RJ. Non-operative management of uncomplicated acute appendicitis: using antibiotics is effective and decreases morbidity. *Evid Based Med*. 2013;18(2):67-68. doi:10.1136/eb-2012-100819 | Comment |
| Steurer J. Akute, unkomplizierte Appendizitis kann primär auch antibiotisch behandelt werden [Acute, uncomplicated appendicitis can also be primarily treated with antibiotics]. *Praxis (Bern 1994)*. 2013;102(6):353. doi:10.1024/1661-8157/a001232 | Comment |
| Mikulicic F. Antibiotika-Therapie bei unkomplizierter akuter Appendizitis effektiv und sicher [Antibiotic therapy in uncomplicated acute appendicitis is effective and reliable]. *Praxis (Bern 1994)*. 2012;101(21):1385-1386. doi:10.1024/1661-8157/a001078 | Comment |
| Andersson RE, Schein M. Antibiotics as first-line therapy for acute appendicitis: evidence for a change in clinical practice. *World J Surg*. 2012;36(9):2037-2038. doi:10.1007/s00268-012-1647-4 | Comment |
| Julià D, Gómez N, Codina-Cazador A. Antibiotic treatment for uncomplicated acute appendicitis. *Lancet*. 2012;379(9822):e45. doi:10.1016/S0140-6736(12)60512-1 | Comment |
| Allescher HD. Appendicitis: can immediate antibiotic treatment still be withheld?. *Gastroenterology*. 2012;142(3):666-669. doi:10.1053/j.gastro.2012.01.019 | Comment |
| Füeßl HS. Bei der unkomplizierten akuten Appendizitis reichen Antibiotika. MMW - Fortschritte der Medizin. 2012;154(11):34-. | Comment |
| DTB Select: 6 \| June 2012. Drug and Therapeutics Bulletin. 2012;50(6):62-5. | Comment |
| De Waele JJ, Blot S. Antibiotic use and delayed source control in acute appendicitis. *Arch Surg*. 2007;142(1):99-100. doi:10.1001/archsurg.142.1.99-b | Comment |
| Van der Peet DL. Acute appendicitis kan ook uitsluitend met antibiotica worden behandeld. Ned Tijdschr Geneeskd. 2006;150:2838-9. Published December 23, 2006. Accessed October 29, 2025. https://www.ntvg.nl/artikelen/acute-appendicitis-kan-ook-uitsluitend-met-antibiotica-worden-behandeld | Comment |
| Kaplan S. Antibiotic usage in appendicitis in children. *Pediatr Infect Dis J*. 1998;17(11):1047-1048. doi:10.1097/00006454-199811000-00019 | Review |
| RICE BH. CONSERVATIVE, NON-SURGICAL MANAGEMENT OF APPENDICITIS. *Mil Med*. 1964;129:903-920. | Review |
| Sychev MD. Vliianie antibiotikov na razvitie i techenie ostrogo appenditsita [Effect of antibiotics on the development and course of acute appendicitis]. *Klin Khir (1962)*. 1978;(4):70. | Case report |
| HARRISON PW. Appendicitis and the antibiotics. *Am J Surg*. 1953;85(2):160-163. doi:10.1016/0002-9610(53)90476-0 | Case series |
| Antibiotic therapy in appendicitis. [in French] NOUV. PRESSE MED. 1980;9(18):1277-1278. Cited in: Embase at https://ovidsp.ovid.com/ovidweb.cgi?T=JS&PAGE=reference&D=emed3&NEWS=N&AN=10148554. Accessed October 30, 2025. | Report not retrieved |
| Milthers E., Baden H., Jensen K. Soap bath as ulcer treatment. Appendicitis and antibiotics. UGESKR. LAEG. 1979;141(9):592-593. Cited in: Embase at https://ovidsp.ovid.com/ovidweb.cgi?T=JS&PAGE=reference&D=emed2&NEWS=N&AN=9112375. Accessed October 30, 2025. | Report not retrieved |
| Dennis. The relationship of elevated temperature and white blood count to antibiotic use in uncomplicated appendicitis. PAS reporter. 1976;14(2). | Report not retrieved |
| Chambers DG, Forsell P. Childhood mortality from acute appendicitis: the impact of antibiotics. *Med J Aust*. 1971;2(24):1255-1256. | Report not retrieved |
| Naess K. Appendicitt og bruk av antibiotika. Faren for alvorlige komplikasjoner [Appendicitis and the use of antibiotics. The danger of serious complications]. *Tidsskr Nor Laegeforen*. 1968;88(4):284-285. | Report not retrieved |
| OBANDO R, ALMONTE L, VANOORDT A. ANTIBI'OTICOS Y DRENAJE EN APENDICITIS AGUDA [ANTIBIOTICS AND DRAINAGE IN ACUTE APPENDICITIS]. *Acad Peru Cir*. 1963;16:213-226. | Report not retrieved |
| COLE WR, BERNARD HR. A reappraisal of the effects of antimicrobial therapy on the course of appendicitis in children. *Am Surg*. 1961;27:29-32. | Report not retrieved |
| ROVIRA'LTA E. Las apendicitis antibióticas en el niño. II. Diagnóstico, tratamiento; conclusiones [Antibiotic appendicitis in children. II. Diagnosis; therapy, conclusions]. *Rev Esp Pediatr*. 1959;15(87):333-342. | Report not retrieved |
| ROVIRALTA E. Las apendicitis antibióticas en el nino [Antibiotic appendicitis in the child]. *Rev Esp Pediatr*. 1959;15(85):57-71. | Report not retrieved |
| SEDOVA NB. Lechenie antibiotikami bol'nykh ostrym appenditsitom v stadii infil'trata [Antibiotic therapy in acute infiltrative appendicitis]. *Sov Med*. 1958;22(10):39-45. | Report not retrieved |
| PASCHOUD H. Traitement abortif antibiotique lors d'appendicites aiguës [Abortive treatment with antibiotics in acute appendicitis]. *Gastroenterologia*. 1958;89(3-4):173-179. | Report not retrieved |
| LOZANO RH, BERMUDEZ A Jr. Apendicitis aguda y consideraciones sobre el uso racional de los diferentes agentes quimioterápicos y antibióticos; análisis estadísticos y resultados en ciento cincuenta y un casos [Acute appendicitis & consideration of the rational use of different chemotherapeutic agents & antibiotics; statistical analysis & results in 151 cases]. *Rev Med Hondur*. 1957;25(3):94-105. | Report not retrieved |
| COHN R, RELFE JD, FIRPO J. The effects of the antibiotics on the treatment of appendicitis. *Stanford Med Bull*. 1953;11(4):230-231. | Report not retrieved |
| FOWLER EF, BOLLINGER JA. Appendicitis, antibiotics, and surgical drainage. *Am Surg*. 1953;19(9):858-866. | Report not retrieved |
| MORODER J, LOPEZ F. Apendicitis y antibióticos [Appendicitis and antibiotics]. *Prensa Med Argent*. 1952;39(36):2137-2139. | Report not retrieved |
| KEYES EL, HAWK BO. Appendicitis and antibiotics: questions and answers. *Med Bull St Louis Univ*. 1952;4(5):96-98. | Report not retrieved |
| SINHA HN. Aureomycin in appendicitis. *J Indian Med Assoc*. 1951;20(11):415. | Report not retrieved |
| CARCASSONNE F, CARCASSONE M. Les appendicites aiguës pénicillinées [Penicillin effects in acute appendicitis]. *Rev Med Moyen Orient*. 1951;8(2):194-197. | Report not retrieved |
| RIGHINI A. L'antibiosi nell'appendicite acuta dell'infanzia; considerazioni clinico-statistiche su 155 casi [Antibiotics in acute appendicitis in children; clinico-statistical considerations on 155 cases]. *Riv Clin Pediatr*. 1950;48(12):759-762. | Report not retrieved |
| VELIKORETSKII AN, KRESTOVNIKOVA GS. Penitsillinoterapiia pri ostrom appenditsite [Penicillin therapy in acute appendicitis]. *Sov Med*. 1950;10:8-10. | Report not retrieved |
| PARTURIER G, BECQUET R. Appendicite et pénicilline [Appendicitis and penicillin]. *J Sci Med Lille*. 1949;67(13):233-237. | Report not retrieved |
| SEEAR-JENSEN T. Akut appendicitis behandlet met penicillin og sulfathiasol [Acute appendicitis treated with penicillin and sulfathiasole]. *Ugeskr Laeger*. 1948;110(36):1023. | Report not retrieved |
| LANDESMAN W. Acute appendicitis treated with penicillin. *U S Nav Med Bull*. 1946;46:1474. | Report not retrieved |

**Table S3.** Grading of Recommendations Assessment, Development, and Evaluation (GRADE) Analysis

| **Certainty assessment** | | | | | | | **№ of patients** | | **Effect** | | **Certainty** | **Importance** |
| --- | --- | --- | --- | --- | --- | --- | --- | --- | --- | --- | --- | --- |
| **№ of studies** | **Study design** | **Risk of bias** | **Inconsistency** | **Indirectness** | **Imprecision** | **Other considerations** | **Antibiotics** | **control** | **Relative (95% CI)** | **Absolute (95% CI)** |  |  |
| **Initial treatment success (RCT-only analysis)** | | | | | | | | | | | | |
| 2 | randomised trials | serious^a^ | serious^b^ | not serious | not serious | none | 147/156 (94.2%) | 144/155 (92.9%) | **RR 1.03** (0.92 to 1.15) | **28 more per 1,000** (from 74 fewer to 139 more) | ⨁⨁◯◯ Low^a,b^ | IMPORTANT |
| **Recurrence of appendicitis** | | | | | | | | | | | | |
| 3 | randomised trials | serious^c^ | serious^d^ | not serious | very serious^e^ | none | 44/196 (22.4%) | 29/170 (17.1%) | **RR 1.38** (0.68 to 2.80) | **65 more per 1,000** (from 55 fewer to 307 more) | ⨁◯◯◯ Very low^c,d,e^ | IMPORTANT |
| **Appendectomy during long-term follow-up** | | | | | | | | | | | | |
| 3 | randomised trials | serious^c^ | not serious | not serious | very serious^e^ | none | 71/225 (31.6%) | 67/212 (31.6%) | **RR 0.98** (0.66 to 1.47) | **6 fewer per 1,000** (from 107 fewer to 149 more) | ⨁◯◯◯ Very low^c,e^ | IMPORTANT |

**CI:** confidence interval; **RR:** risk ratio

#### Explanations

a. Most information comes from studies at moderate risk of bias.

b. I² = 54%, and no plausible a priori hypothesis was available to explain the observed heterogeneity.

c. Most information comes from studies at moderate risk of bias. Sensitivity analysis excluding the high-risk study showed no meaningful change in the effect estimates.

d. I² = 55%, and no plausible a priori hypothesis was available to explain the observed heterogeneity.

e. The MCID was pre-specified as RR 0.75/1.25. Although the point estimate suggested no significant effect, the confidence interval spanned both possible clinically important harm and benefit.
